# Arms of larval seastars of *Pisaster ochraceus* provide versatility in muscular and ciliary swimming

**DOI:** 10.1371/journal.pone.0213803

**Published:** 2019-03-14

**Authors:** Sophie B. George, Richard R. Strathmann

**Affiliations:** 1 Biology Department, Georgia Southern University, Statesboro, Georgia, United States of America; 2 Friday Harbor Laboratories, University of Washington, Friday Harbor, Washington, United States of America; National Taiwan Normal University, TAIWAN

## Abstract

Larval swimming with cilia, unaided by muscles, is the presumed ancestral condition for echinoderms, but use of muscles in swimming has evolved several times. Ciliation and musculature of the arms of brachiolaria-stage larvae in the family Asteriidae provide unusual versatility in the use of muscles in swimming. The muscles affect swimming in two different ways. (1) Contraction of muscles moves the arms, propelling the larva. (2) Contraction of muscles changes orientation of the arms, thereby changing direction of ciliary currents and direction of swimming. New observations of the brachiolaria of the asteriid seastar *Pisaster ochraceus* demonstrate more versatility in both of these uses of muscles than had been previously described: the posterolateral arms stroke in more ways to propel the larva forward and to change the direction of swimming, and more pairs of the arms point ciliary currents in more directions for changes in direction of swimming. Morphology of brachiolariae suggests that these uses of muscles in swimming evolved before divergence of the families Stichasteridae and Asteriidae within forcipulate asteroids. This versatile use of muscles for swimming, both alone and in combination with ciliary currents, further distinguishes the swimming of these brachiolariae from swimming by larvae of other echinoderms and larvae of acorn worms in the sister phylum Hemichordata.

## Introduction

Ciliary swimming without the aid of muscles is the general and presumably ancestral case for the feeding larvae of echinoderms [1]. Use of muscles in larval swimming, known to occur in the seastar family Asteriidae, is striking because use of muscles in larval swimming evolved rarely in the phylum Echinodermata. Muscles of echinoderm larvae supplement swimming in two ways. One way is for muscles to create a current by moving part of the larval body. Another way is for muscles to redirect ciliary currents by changing the form of the larval body.

Both uses of muscles occur in swimming by the brachiolaria larvae in the family Asteriidae and perhaps in some other families in the order Forcipulatida. Brachiolaria larvae develop 5 pairs of larval arms (the preoral, anterodorsal, postoral, posterodorsal and posterolateral arms). The longest of the 5 pairs of arms are the posterolateral arms, which in asteriids can grow to over 2 mm long [2]. The other pairs of larval arms extend to half or less than half the length of the posterolateral arms. Two modifications of the larval arms of these brachiolariae confer unusual versatility in the use of muscles in swimming. A modification that antedates the common ancestor of forcipulatid seastars is muscles that change orientation of the arms. These muscles are well developed in asteriids; muscle strands that arise from each arm extend considerable distances under the body wall, occasionally crossing or connecting with strands from other arms [1,3]. The brachiolariae of asteriids can move the posterolateral arms to rotate while suspended vertically [3,4]. A second modification is a change in shape of cells in the arms, so that the arms are more evenly ciliated, and a change in direction of beat of the cilia, so that the ciliary current is directed toward the tips of the arms rather than across the arms [1,5]. This second modification appears to have evolved within the order Forcipulatida. It has been demonstrated only for asteriid brachiolariae but is expected to occur in some other forcipulatid brachiolariae because of the shape and length of the larval arms [6]. Brachiolariae with these modifications can reverse direction of swimming by pointing a ciliary current, directing the arms anteriorly instead of posteriorly [1,5]. Gemmill [5] observed that movements of the larva’s processes (arms) affected swimming in diverse ways but gave few details on the movements, reporting that the arms can be moved in all directions, are employed for oblique, horizontal, and downward movements, and serve for both progression and direction of progression.

The objective of this study was to illustrate the varied ways that the brachiolaria larvae of the asteriid *Pisaster ochraceus* both move their arms in strokes for swimming and direct their arms to point ciliary currents. The versatility observed in brachiolariae of *P*. *ochraceus* and expected for brachiolariae of some other forcipulate asteroids contrasts with the limited use of muscles in swimming by other echinoderm larvae. In the class Echinoidea, some echinoplutei change orientation of arms, which changes direction of ciliary currents and presumably thereby changes the swimming speed [7]. In the class Asteroidea, contraction of dorsal muscles of bipinnariae bends the larval body, redirecting ciliary currents so that the larva turns toward its dorsal side [1,5,8]. A less common role of muscles in swimming is to produce a current by changing shape of the larval body. Bipinnariae of some species of *Luidia* (order Paxillosida) swim by undulations of the unusually long anterior part of the larval body [9]. Those are the known uses of muscles in swimming by larvae of other echinoderms. Moreover, muscles are not used in swimming by larvae in the Hemichordata [10,11,12]. The Hemichordata and Echinodermata are inferred to be sister groups with similarities and inferred homologies in their feeding larval stage [13]. Here we describe previously unreported movements and positions of arms of an asteriid brachiolaria that affect it’s swimming in varied ways.

## Materials and methods

### Obtaining larvae

Permission to collect specimens at the two field sites was provided by the director at Friday Harbor Laboratories, University of Washington.

The brachiolariae used for these observations were reared from embryos obtained from several females and males collected from Cantilever Point (48° 32’ 46”N, 123° 0’ 46” W, June 2014) and Point Caution (48° 56’ 31”N, 123° 02’ 44” W, May 2017), San Juan Island, Washington. Individuals were spawned, eggs fertilized, and embryos distributed into 3.5-liter glass jars containing 30‰ seawater following published methods [4,14,15]. Jars were placed in a sea table with continuous flowing seawater from San Juan Channel that varied between 11–15°C. As the larvae developed, they were fed *Dunaliella tertiolecta* and *Rhodomonas* sp. (1000–5000 cells/ml) [2,16] and kept in suspension with a system of swinging paddles [14, 17]. Before adding food to culture jars, cultures were cleaned once a week by siphoning 80–90% of the seawater out and replacing it with 0.45μm filtered seawater. Larvae were maintained at one larva per ml (2014) or one larva per 10 ml (2017).

### Observations

When larvae reached the brachiolaria stage, they were removed from jars, placed on depression slides in the same seawater, and allowed to swim for several minutes before videotaping. Ages of the videotaped brachiolariae ranged from 40 to 84 days. In both years, behavior was recorded with a video camera attached to a compound microscope and images captured with BTV pro software.

Video clips of three larvae provided especially clear views of behavior and were used for the figures. For figures, video clips were first converted to MPEG4 format and then exported as frames to Adobe Photoshop. For each figure of larvae on the slide, the positions of frames were fixed during intervals when the slide was not moved, so that changed positions of larvae indicate their motion. Observations of larvae on a slide provided informative magnification and permitted, perhaps stimulated, diverse behavior. Speeds can be calculated from position, scale lines, and numbers for elapsed time, but nearby surfaces on the depression slide affect speed of the larvae and paths of particles. Temperatures on the depression slide were not controlled. Room temperature was between 20 and 23°C.

Limited observations were also made of larvae in acrylic cylinders that were 45 cm tall and 9 cm in diameter and placed in a sea table with continuous flowing seawater. The setup is described by Bashevkin et al. [4]. Haloclines were set up with 20‰ sea water at the top and 30‰ at the bottom of the cylinder by slowly gravity feeding the 20‰ filtered sea water through a tube to 15 cm high in the cylinder. This was followed by 30‰ filtered seawater gravity fed below the 20‰ sea water up to the 40 cm mark in the cylinder. The water temperature in the cylinders was between 11 and 12°C. One hundred to 250 brachiolaria larvae were slowly gravity fed to the bottom of cylinders through 167cm long 0.32 x 0.64 cm tubes. Brachiolariae were allowed to swim for 90 minutes before observations and pictures were taken. The picture presented in this study represents what was seen in cylinders with 20‰ seawater at the top and 30‰ seawater at the bottom.

## Results

### Turning with strokes of the posterolateral arms

**Fig 1:**
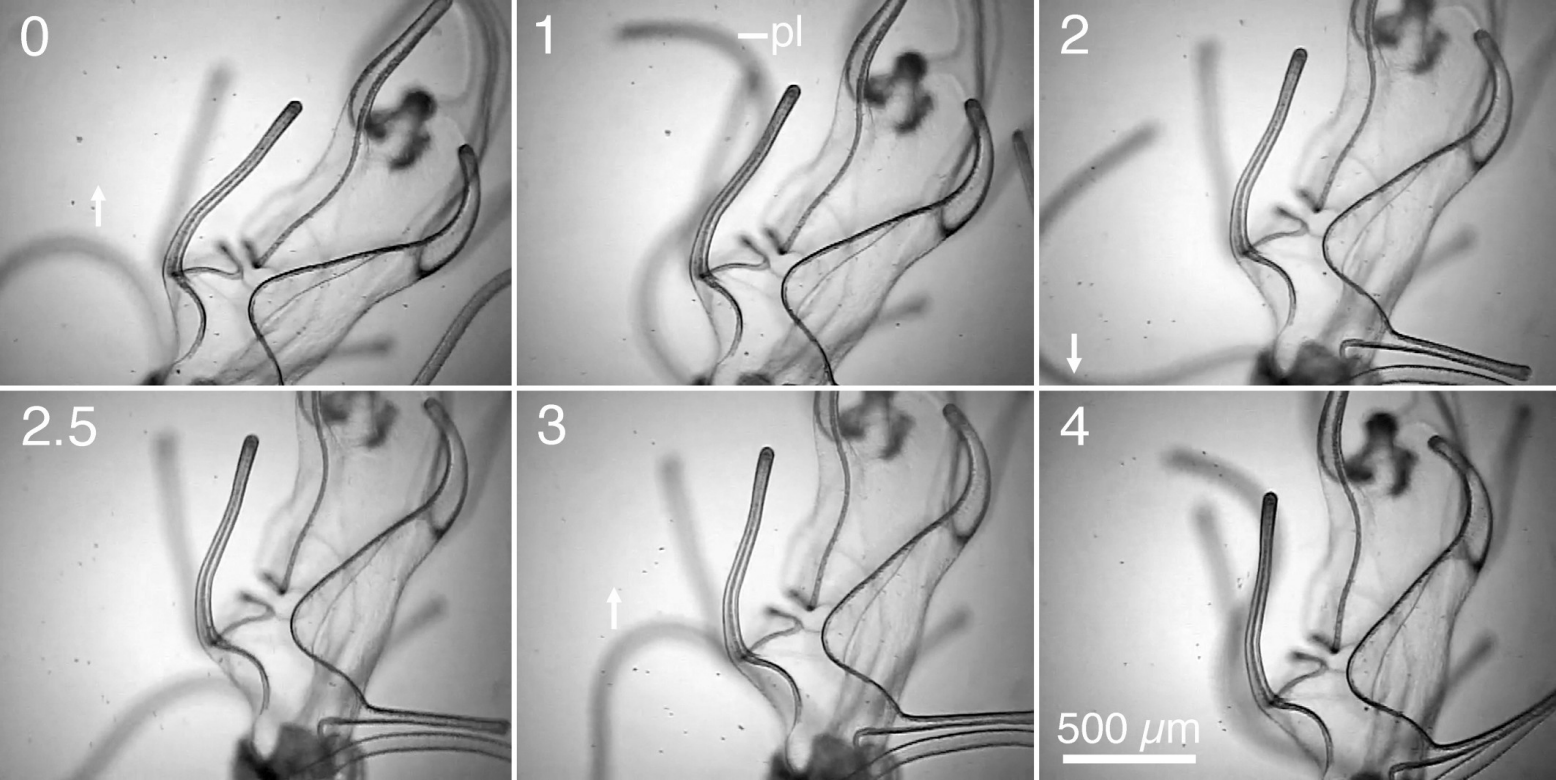
Turning ventrally by strokes of a posterolateral arm. The right posterolateral arm (pl) is directed ventrally and strokes. The left posterolateral arm is directed dorsally. The larva turns ventrally (a counterclockwise rotation as viewed toward its left side). Numbers at the upper left of the frames are time in seconds. Arrows indicate the direction of motion of the right posterolateral arm.

**Fig 2:**
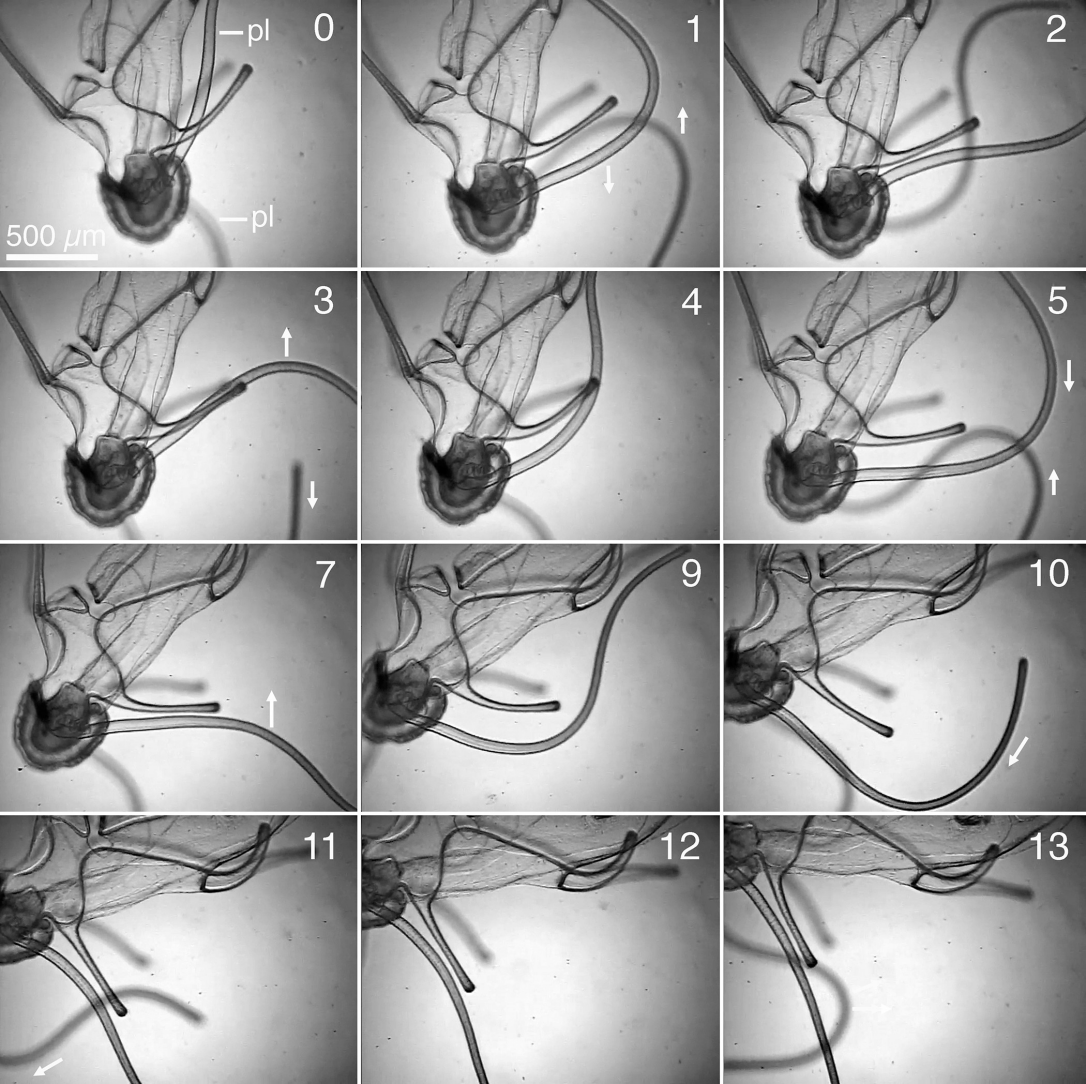
Turning dorsally by strokes of the posterolateral arms. The posterolateral arms (pl) are directed dorsally. The larva turns dorsally (a clockwise rotation as viewed toward its left side). Numbers at the upper right of the frames are time in seconds. Arrows indicate direction of motion of the posterolateral arms. From 0 through 10 seconds both posterolateral arms stroke. From 11 to 13 seconds the left posterolateral arm moves little while the right posterolateral arm continues strokes.

A brachiolaria larva, age 49 days, turned toward its dorsal or ventral side by strokes of both posterolateral arms or by strokes of just one posterolateral arm while the arm on the other side moved less. For a larva viewed from its left side, the turn was by a counterclockwise rotation toward the ventral side (Fig 1) or clockwise rotation toward the dorsal side (Fig 2). In these frames from a video recording, the left posterolateral arm is in the plane of focus and the right posterolateral arm is more distant from the plane of focus.

In the ventrally directed turn in Fig 1, the right posterolateral arm was directed ventrally and completed a full stroke cycle between 1 and 4 seconds. During this interval, the right posterolateral arm was directed dorsally and initially moving, was then out of the frame at 1 second, and moved little between 2 and 4 seconds. Six seconds later, the left posterolateral arm had moved so that both arms were directed dorsally, and the brachiolaria began to turn dorsally (not shown in figures). Fig 2 illustrates the dorsally directed turn, beginning thirteen seconds after Fig 1 ends. The two posterolateral arms began strokes in opposite directions at 0 and again at 4 seconds. The strokes were thus initially in opposite phases. By 11 to 13 seconds, however, the left posterolateral arm had ceased strokes while the right posterolateral arm continued strokes.

In these strokes the bend in the posterolateral arm travels from base to tip. Paths of particles indicated that the moving posterolateral arms produced a current away from the larva. The larva rotated 90° in 13 seconds while the posterolateral arms made three strokes. The rate of turning from arm strokes may have been affected by the larva’s confinement in a narrow space and in a horizontal orientation. A video that includes these strokes and other strokes of arms described below is in the supplementary materials for Bashevkin et al. [4] (www.intres.com/articles/suppl/m542p123_supp/original/M_11563_Suppl_vid.mp4), but without analysis or discussion of the arm movements.

In the brachiolaria stage, the ciliary beat produces a current from the base toward the tip of each arm. All of the 10 larval arms can therefore influence direction of motion by pointing a ciliary current. Strokes of posterolateral arms and orientation of other arms occurred in varied combinations during turning. In another sequence a postoral arm stroked as well as the posterolateral arms. During the turns in Fig 1 and Fig 2, however, the preoral, anterodorsal, and postoral arms were directed anteriorly and the posterodorsal pair extended out from the larval body. The ciliary currents from these arms could have affected the rates of turning in Fig 1 and Fig 2, but their fixed directions indicate that currents on these arms did not control the directions of turning.

### Swimming forward by a fish-tail movement of the posterolateral arms

**Fig 3:**
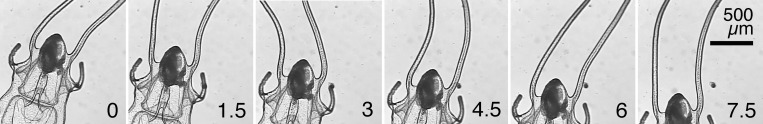
Swimming forward with side-to-side strokes of the posterolateral arms. The posterolateral arms are directed posteriorly and move from side to side like a fish’s tail. Numbers at the lower right of the frames are time in seconds.

Brachiolaria larvae can swim forward by moving the posterolateral arms from side to side like a fish’s tail. In Fig 3A brachiolaria, age 40 days, swam forward while completing a stroke cycle, back and forth, in about 6 seconds. The side-to-side movement of the posterolateral arms was often seen when these younger brachiolaria larvae were propelling themselves upwards towards the surface of the water column.

Ciliary currents are directed toward the tips of the posterolateral arms at this stage [1] and also enhance forward swimming. The postoral and posterodorsal arms were directed posteriorly, and ciliary currents along these arms could also have enhanced forward swimming. The preoral and anterodorsal arms (not within the video frames in Fig 3) were curved and pointed laterally away from the larval body and would have little effect on forward swimming.

### Swimming with laterally directed strokes of the posterolateral arms

**Fig 4:**
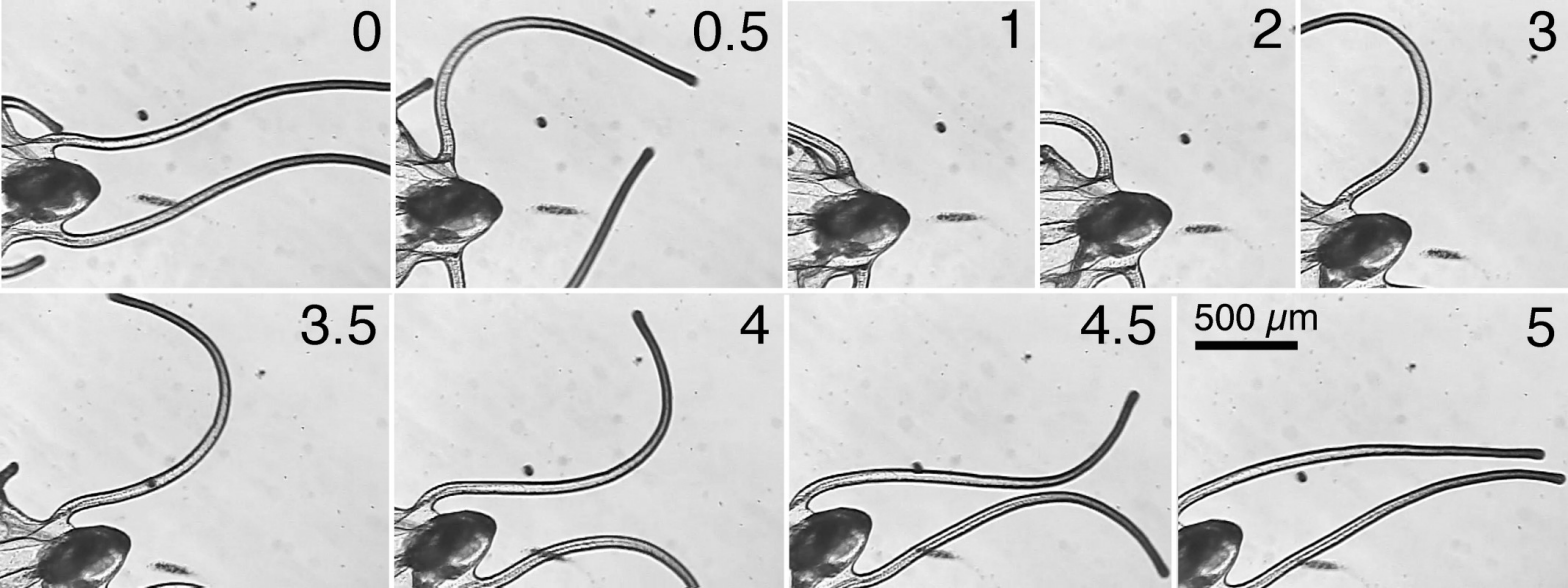
Lateral strokes of the posterolateral arms. The posterolateral arms move apart laterally and then together medially. Numbers at the upper right of the frames are time in seconds.

The same brachiolaria also moved its posterolateral arms apart, laterally and anteriorly, and then together, medially and posteriorly (Fig 4). A cycle of this stroke did not result in much forward motion. During these strokes the bend in the arm began proximally and moved distally. Lacalli [3] also observed this stroke of posterolateral arms and said that this action causes them to rotate on axis when the larvae are suspended vertically in the feeding posture. The transitions in movement of the posterolateral arms from the side-to-side movement (as in Fig 3) to anterior to posterior movement (as in Fig 4) and then back to side to side were immediate and without pause.

### Turning by pointing the ciliary currents of postoral and posterodorsal arms

**Fig 5:**
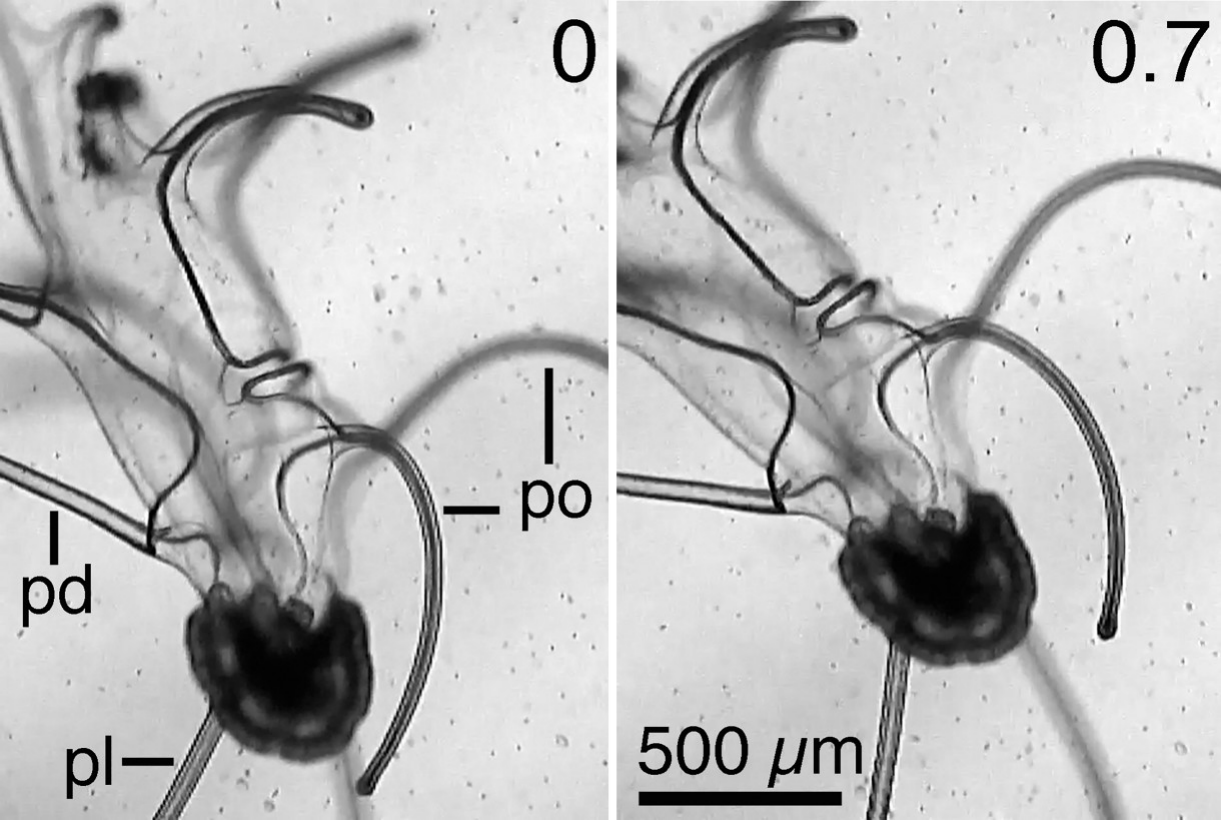
Pointing ciliary currents by pointing arms. The posterodorsal arms (pd) point anteriorly and the postoral arms (po) point posteriorly. Ciliary currents that run from base to tip of arm turned the larva dorsally (a counterclockwise rotation as viewed toward the larva’s right side). The posterolateral arms (pl) pointed posteriorly and did not stroke. Numbers at the upper right of the frames are times in seconds.

A brachiolaria larva, age 84 days, turned by pointing arms to direct the ciliary currents along the arms. The larva turned while all arms were held in fixed positions (Fig 5, S1 Video). The postoral arms pointed posteriorly and the posterodorsal arms pointed anteriorly, directing ciliary currents that turned the larva dorsally (rotated counterclockwise as viewed from the larva’s right side). It was previously observed that the brachiolariae can reverse direction of swimming by pointing arms and their currents anteriorly [1]. The brachiolariae of *P*. *ochraceus* are versatile in pointing arms to point currents in different directions as well as versatile in moving the especially long posterolateral arms.

### Swimming in a larger volume of water

**Fig 6:**
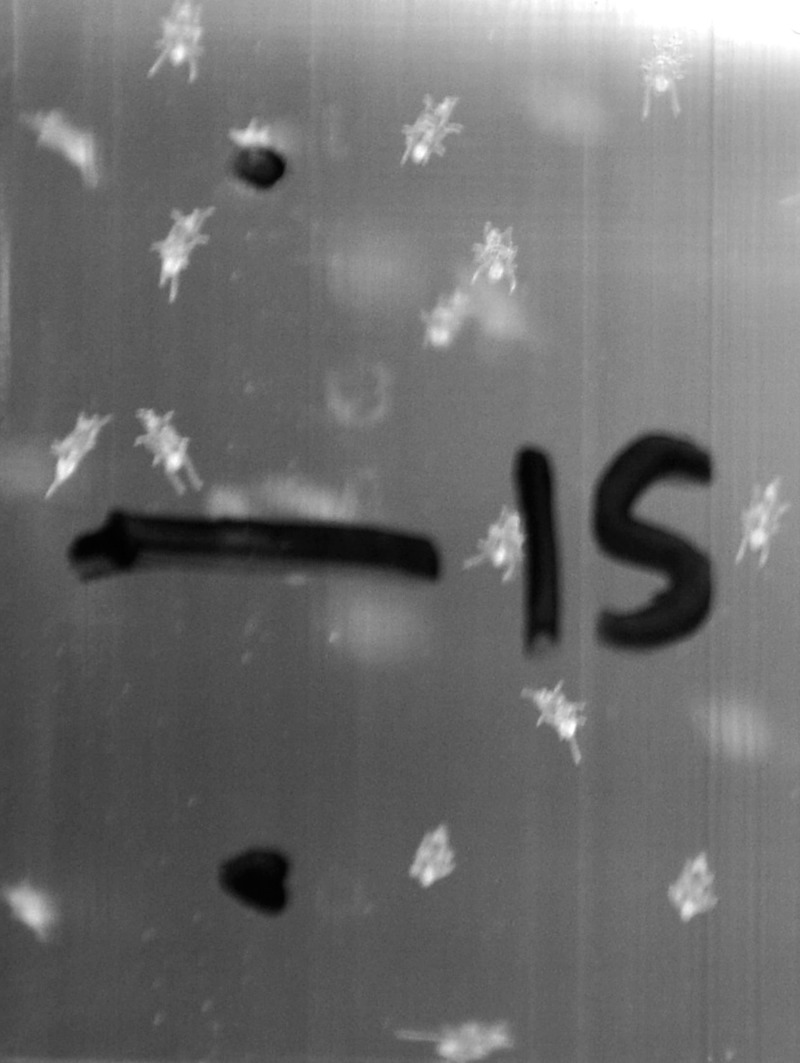
Brachiolariae swimming in a column of water. The larvae had been introduced at the bottom of a column 45 cm tall. The line on the column is 15 cm from the top, and each dot is 1 cm away from the line. The dots and line mark the position of a halocline with 30‰ below and 20‰ above. Most but not all larvae are swimming upward, with anterior ends toward the top of the figure. Posterolateral arms of most individuals are pointed posteriorly. Posterolateral arms of two individuals (at lower right of the figure) are directed anteriorly.

The larvae in Figs 1–5 were confined on their sides in a small volume of water. The larvae also held their arms in a variety of positions when free to swim in any direction in a large volume of water. The larvae in Fig 6 were in the column used by Bashevkin et al. [4]. Posterolateral arms of most individuals pointed posteriorly, a position that increases speed by pointing a ciliary current [1,2] and may also be enhanced by strokes of the arms. Posterolateral arms of a few individuals were pointed anteriorly, a position that could reverse direction or change orientation.

## Discussion

Swimming of larvae of asteroids and other echinoderms changes during development and differs with morphology [18,19]. The arms of brachiolariae of *Pisaster ochraceus* confer capabilities not present in the preceding bipinnaria stage or in the brachiolariae of other orders of asteroids. Advanced brachiolaria larvae of *P*. *ochraceus* have a suite of complex, precise, and well-coordinated swimming behaviors that aid directionality of swimming. Speed may be enhanced by currents generated by the arms as well as by use of arms in maintaining a direction. These larvae can turn and switch directions in a few seconds by strokes of the posterolateral arms or by pointing ciliary currents with either the posterolateral arms alone or with the postoral and posterodorsal arms in combination. The posterolateral arms can sweep through the water synchronously or asynchronously to complete turns in either the dorsal or ventral direction. The side-to-side movement of the very long posterolateral arms adds to ciliary beat as a means of forward swimming. The posterolateral arms can grow to be almost as long as the rest of the larval body. The larvae can also change direction by pointing the arms in different combinations to point the ciliary currents along the arms.

Pointing the ciliary currents along the arms is reported for two other species in the family Asteriidae, *Asterias rubens* [5] and *Pycnopodia helianthoides* [1]. Pointing arms provides another way for advanced brachiolariae to maintain their orientation or their position at a particular depth. Long, thin, and non-tapering arms also occur in *Coscinasterias calamaris* (family Asteriidae) and *Stichaster australis* (family Stichasteridae) [6]. Brachiolariae with similar arms that are used in similar ways are therefore expected to occur in other species in the family Asteriidae and in at least one other family in the order Forcipulatida. Morphology suggests an origin of this kind of arm within forcipulate asteroids before the divergence of ancestors of the Asteriidae and Stichasteridae [20]. A precursor to this evolutionary innovation is muscles that move arms. Movement of arms is widespread in late stage asteroid larvae, although in other orders, so far as is known, the arms are less mobile. Also, in the development of larvae in other groups of asteroids, so far as is known, the cilia continue to form a band along the edges of the arm, with cilia beating perpendicular to the band rather than toward the tip of the arm. The behaviors observed here appear to be an evolutionary innovation unique to a clade within the order Forcipulatida.

The versatility in muscular and ciliary swimming that is conferred by this evolutionary innovation is expected to be adaptive. Diverse larvae respond to stimuli in order to swim at specific depths that offer advantages for feeding where food is abundant, for faster development where water is warmer, for escape from predators, or for transport in currents; at late stages larvae from benthic adults swim to leave the plankton and find a suitable habitat at which to settle and cease swimming [21].

Asteroid larvae are no exception. Asteroid larvae have been found exclusively in the upper mixed layer of a shallow embayment [22]. Brachiolariae of *P*. *ochraceus* lingered at a layer of water with a higher concentration of algal cells [4]. Exposure to low salinities for several days during development of *P*. *ochraceus* resulted in shorter posterolateral arms relative to body length [2] and also affected their movement through a salinity gradient and a layer with a high concentration of algal cells [4]. Asteroid larvae can adjust their speed of swimming in the bipinnaria stage before they develop arms, presumably by modulating ciliary beat [8], and as one means of changing direction they contract dorsal muscles, which bend the larval body [1,5,8]. The extent to which the larval arms in brachiolariae of asteriids increases performance in swimming will be determined by comparisons with swimming by bipinnariae, which have not yet developed the arms, and comparisons with swimming by brachiolariae in other orders of asteroids. Measures of performance include both speed and maneuver. As an example, swimming speed is greater at higher temperature; blastulae and plutei of echinoids adjust their speed upward by widening their helical path at higher temperatures and narrowing the helix at lower temperatures [23,24], but that adjustment was not evident for a bipinnaria [25]. The size of bipinnariae and brachiolariae will permit video recording of their behavior in a volume of water large enough to avoid wall effects and admit a variety of conditions.

A larva has the challenges of changing its direction of swimming when it encounters objects and of maintaining its direction of swimming when shear turns its body [19,26–28]. The ability of a brachiolaria to move its arms to turn or rotate may help it counter the effects of shear. Swimming of different asteroid larvae in shear and turbulence can be compared in the laboratory, as has been done with larvae of echinoids and mollusks [19,26,29–33]. Such observations will demonstrate the extent to which an evolutionary innovation in larval arms has enhanced performance in swimming. Brachiolariae of asteriids move their arms in response to a shaking [5], which suggests that the arms could also be used to sense shear.

Evolutionary innovations in structure of arms of brachiolariae that occurred in the order Forcipulatida have provided unusual versatility in muscular and ciliary swimming. It is certainly possible that the larvae use the arms in more ways than were recorded here. A testable hypothesis is that these arms provide capabilities beyond those in the preceding bipinnaria stage and beyond those of brachiolariae in other orders of seastars.

## Supporting information

S1 Video Video footage of 84 day-old brachiolaria larva of *Pisaster ochraceus* swimming. (MOV)
